# Negative childbirth experience in Dutch women: A socio-ecological analysis of individual, interpersonal, and organisational factors from the birth experience study

**DOI:** 10.1016/j.heliyon.2024.e41254

**Published:** 2024-12-15

**Authors:** Tamool A.S. Muhamed, Viola Angelini, Laura Viluma, Hazel Keedle, L. Lilian Peters

**Affiliations:** a University of Groningen, Faculty of Economics and Business, Department of Economics, Econometrics, and Finance, Nettelbosje, 9700, AV, Groningen, the Netherlands; b University of Groningen, University Medical Centre Groningen, Primary and Long-term Care, Section Midwifery Science, Hanzeplein 1, 9713, GZ, Groningen, the Netherlands; c Amsterdam UMC, Location Vrije Universiteit Amsterdam, Midwifery Science, De Boelelaan 1117, 1081 HV, Amsterdam, the Netherlands; d Western Sydney University, School of Nursing and Midwifery, Locked Bag 1797, Penrith, NSW, 2751, Sydney, Australia

**Keywords:** Socio-ecological model, Childbirth, Decision making, Respect, Continuity of care, Midwifery

## Abstract

**Background:**

Negative childbirth experience detrimentally impacts women's mental well-being, potentially leading to delaying future pregnancies, and an increased likelihood of requesting caesarean births.

**Aim:**

To examine differences between women who reported positive and negative childbirth experience and detangle the complexity of negative childbirth experience by building a socio-ecological model that includes individual, interpersonal, and organisational factors.

**Methods:**

We conducted the Birth Experience Study Netherlands (BESt-NL) survey in 2022 with two languages versions (Dutch-English), and incorporated validated measures, such as Mothers' autonomy in decision-making, the Mothers On Respect index, and the Nijmegen Continuity of Care questionnaire. We employed socio-ecological modelling of individual (e.g., sociodemographic, ethnicity, parity, adverse mental health, interpersonal (e.g., autonomy in decision-making, respect, partner support), and organisational factors (e.g., place of birth, continuity of care). We defined negative childbirth experience using the valid Childbirth Experience Questionnaire 2.0. We applied multivariable logistic regression to examine associations between those factors and negative childbirth experience.

**Findings:**

In total, (N = 1141) women were included in the BEST-NL study population, and 25 % of women (N = 285) experienced negative childbirth. Higher percentages were observed for non-Dutch ethnicity, preterm births, pregnancy complications, non-spontaneous births, adverse mental health, obstetrician-led care, and low autonomy, respect, social support, and continuity of care. Upon modelling, significant associations emerged i.e., education; or diminished i.e., place of birth; leaving robust associations in preterm, non-spontaneous birth, and adverse mental health, and inverse associations in high autonomy, respect, social support, and continuity of care.

**Conclusion:**

Socio-ecological modelling untangled the complexity of negative childbirth experience. This study recommends fostering efforts toward women with prenatal mental health conditions and migrants, emphasises the importance of high autonomy, respect, and continuity in high-quality intrapartum care, and highlights the positive impact of midwife-led care in reducing negative childbirth experience likelihood.

## Highlights

• Negative childbirth experience is linked to delayed pregnancies and caesarean birth.
• The first study to design socio-ecological model in intrapartum healthcare research.
• Mode of birth, autonomy, respect, and continuity of care exhibited robust association.
• Recommendation for prenatal mental health screening.
• Recognizing the benefits of midwife-led care health system.

## 1 Introduction

Childbirth is a central event in women's lives, representing a transformative experience that spans a wide spectrum. On one end, it can be a favourable and empowering experience, while on the other, it can be negative and traumatic [1,2]. The dynamic aspect of childbirth is characterised by interlinked subjective psychological and physiological processes [3]. While positive childbirth experience can provide women with high self-esteem and well-being [4], a negative childbirth experience can exert profound and deleterious effects on women's self-esteem, self-efficacy, and overall mental well-being, which could extend beyond the labour and birth period [5,6]. It can lower quality of life, and self-rated health, also leaves lasting memories of pain, initiates the onset of posttraumatic stress disorder (PTSD), and contributes to complications in maternal-infant bonding [1,7]. Due to the emergence of a persistent fear of childbirth; negative childbirth experience influences a mother's subsequent reproductive decisions, possibly leading to choosing to postpone or entirely forgo a subsequent pregnancy to reduce the chances of a recurrence of such an experience [6]. Those with a negative childbirth experience had their subsequent child 4.2 years later, in contrast to 2.4 years later for those with a positive experience [6], and it has been associated with an elevated likelihood of requesting a caesarean birth [3,6].

Even with the World Health Organisation (WHO) framework for ensuring high-quality intrapartum care that emphasises concepts such as respectful care, autonomy, effective communication, and the consistent availability of competent staff [4], a recent national Dutch survey conducted in 2022 with 12,239 women revealed the regular occurrence of disrespectful and abusive encounters during labour and childbirth. The most frequently reported forms of disrespect and abuse were related to 'lack of choices' (39.8 %), followed by 'lack of communication' (29.9 %), 'lack of support' (21.3 %), and 'harsh or rough treatment/physical violence' (21.1 %). Primiparity (nulliparous) and migrant background emerged as risk factors for experiencing disrespect and abuse [8].

Therefore, we aimed to elucidate the occurrence of negative childbirth by examining differences between women who reported positive and negative childbirth experience by using the valid Childbirth Experience Questionnaire 2.0. Additionally, we innovatively built socio-ecological model, aiming to examine factors associated with negative childbirth experience. We expanded the included factors by incorporating results from the previous literature and recommendations from the WHO framework of high-quality intrapartum care. These additional factors included measures of autonomy, respect, and continuity of care.

Socio-ecological models acknowledge the embedded nature of individuals within broader social systems, delineating the connected dynamics between individuals and their environments that shape health outcomes [9,10]. These models include individual factors (demographics, biomarkers, lifestyle behaviour, and psychology), interpersonal factors (formal and informal social networks and social support systems), and organisational factors [9].

At the individual level, there are many factors that contribute to negative childbirth experiences such as education level, age, ethnicity and parity [3,6,8]. Single women are more likely to have negative experiences [11]. The Lesbians, Bisexual, Transgender, and Queer (LBTQ) community often experience higher childbirth-related anxiety [12]. Preterm birth increases negative perceptions [6]. Pregnancy complications impact the mode of birth and postpartum care [13]. Instrumental vaginal and caesarean births predict negative experiences [3]. Unhealthy behaviours like smoking, alcohol, and substance use harm gestational age and maternal/neonatal health [14]. Pre-existing mental health disorders correlate with lower education, single status, fewer antenatal visits, less weight gain, hospitalization during pregnancy, and longer postpartum hospital stays [15].

At the Interpersonal level, women who receive support from partners, social circles, and healthcare providers tend to have positive childbirth experiences [4]. Active involvement of women in decision-making during pregnancy and childbirth significantly influences overall satisfaction with care [16]. Respectful maternity care emphasises women's entitlement, considering their individual needs and preferences, including the right to information, informed consent, autonomy, and dignity [4,17]. High autonomy and respect potentially contribute to positive childbirth experiences [17].

At the organisational level, the Dutch maternity care system is bifurcated into midwife-led care and obstetrician-led care [18]. In midwife-led care, expectant mothers with the absence of complications could choose to undergo childbirth at home or at a birth centre [18]. Birth centres differ from obstetric-led care in hospitals in the absence of labour induction, pharmaceutical pain relief, and instrumental births. When the clinical condition necessitates specialised expertise, surgery, or involves women with severe complications, cases are referred directly to obstetrician-led care [18]. Obstetrician-led care comprises hospital-based midwives (clinical) and residents, under the supervision of an obstetrician and with the assistance of obstetric nurses [18]. Research found that obstetrician-led care has a higher likelihood of negative outcomes [18,19]. In contrast, midwifery-led continuity of care is associated with a positive childbirth experience [[20], [21], [22]], improved health outcomes, enhanced satisfaction and quality of life of women, and reduced healthcare costs [23].

## 2 Methods

### 2.1 Participants and data collection

This cross-sectional study is reported according to Strengthening the Reporting of Observational Studies in Epidemiology Guidelines [24]. We analysed data from the cross-sectional study, the Birth Experience Study Netherlands (BESt-NL). BESt-NL was an online survey where women were recruited through paid advertisements on social media platforms such as Facebook and Instagram. This approach offered decreased expenses, improved participant diversity, and expedited recruitment processes [25]. Additionally, we targeted outreach on websites like Childbirth Network, which is an initiative led by the Midwifery Academy Amsterdam Groningen affiliated with UMC Groningen and Amsterdam UMC [26]. Additionally, we distributed flyers that contained a link to the survey through various Municipal Health Services (Gemeentelijke Gezondheidsdienst, GGD).

**Fig. 1:**
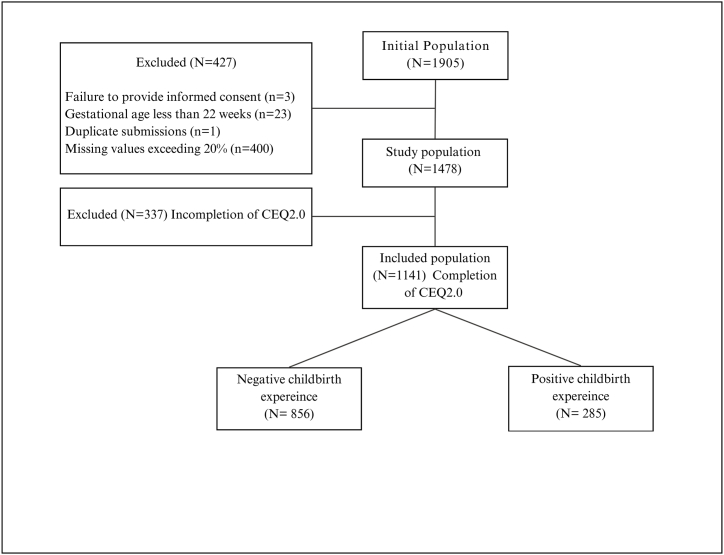
Flowchart of the included population.

The survey included 307 items of individual, interpersonal and organisational factors of maternal and pregnancy-related characteristics and outcomes. The study was conducted from June 2022 to October 31st, 2022, involving women who experienced pregnancy after June 2017 and were over 18 years old. The inclusion criteria consisted of women who were at 22 weeks of gestation or above, gave informed consent, and had no more than 20 % missing values (Fig. 1).

### 2.2 Ethics

The study design and data collection adhered to the principles outlined in the Declaration of Helsinki. As per Dutch regulations, ethical approval was not mandated for this type of research (http://www.ccmo.nl).

### 2.3 Methods

We applied socio-ecological modelling which has been utilised by entities such as the WHO and the Centres for Disease Control and Prevention (CDC) in adopting healthy lifestyle behaviours [9], whereas in maternal health research, it was utilised to reduce sexual assaults [27], understand women's choices in childbirth modes [19], examine healthy lifestyle behaviours of women with overweight [28].

**Fig. 2:**
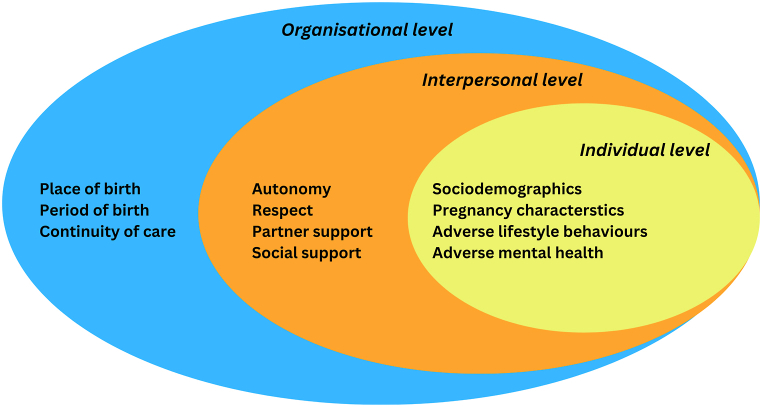
Socio-ecological model to study factors associated with negative childbirth experience.

Building upon this framework, we utilised three levels of influence (Individual, Interpersonal, Organisational). The individual level consisted of sociodemographic, pregnancy-related characteristics such as parity, gestational age, pregnancy complications, and mode of birth, adverse lifestyle behaviours, and adverse mental health. The interpersonal level consisted of partner and social support. We extended the concept to innovatively include measures of autonomy in decision-making, and respect. Finally, we studied organisational factors such as place of birth, period of birth, and we extended the concept to include measures of continuity of care in the Dutch maternal healthcare systems (Fig. 2).

#### 2.3.1 Negative childbirth experience

We defined negative childbirth experience using the Childbirth Experience Questionnaire (CEQ2.0), which was initially developed and validated in Sweden before being adapted into a validated Dutch version [18,29]. This measure comprises 22 items categorised into four domains: 'Own Capacity,' 'Professional Support,' 'Perceived Safety,' and 'Participation.' Among the 22 items, 19 are evaluated using a four-point Likert scale (ranging from 'totally agree' = 4 to 'totally disagree' = 1). In the Dutch CEQ2.0, three items (items 20–22) were appraised using a marking scale from (0–10), akin to the Dutch school marking system, where 0 represents the lowest possible score and 10 is the highest. Furthermore, negatively worded statements and the item concerning labour pain (items 3, 5, 8, 9, and 20) were reversed to ensure that higher scores indicate a more positive experience [18]. Scores are the average item ratings within each subscale [18]. The weighted mean CEQ2.0 score is computed by totalling all subscale scores (own capacity, perceived safety, professional support, and participation) and dividing by four. The scale's theoretical range spans from one to four, with higher scores denoting more positive experiences [18]. For our analysis, we identified the cut-off value for negative childbirth experience as the lowest quartile range of the CEQ 2.0 score, mirroring methodology from a prior study [30].

#### 2.3.2 The online survey for BESt-NL included items on exposures across various levels of influence, namely individual, interpersonal and organisational

##### 2.3.2.1 Individual factors

We categorised maternal age based on the sample's average age, distinguishing between individuals younger or older than 30 years old, then marital status into those in a relationship or single, including divorced or widowed individuals, also, categorised sexual orientation into heterosexual or LGBTQIA+. We categorised education into three levels; low, middle, and high [31], and monthly household income into four categories; low, middle, high, and not willing to report [31]. We categorised ethnicity as Dutch, second-generation migrants (children of one or two migrants), and migrants [31]. In pregnancy characteristics, we included parity (nulliparous/multiparous), gestational age (preterm, full term, late term), pregnancy complications (Yes e.g., diabetes and/or hypertension)/No), and mode of birth (spontaneous vaginal, instrumental vaginal, caesarean section (CS) elective, CS emergency). Lastly, we asked binary questions of adverse mental health; the existence of prenatal mental health disorder i.e., depression or anxiety during two years before or during pregnancy, and adverse lifestyle behaviours; smoking, alcohol consumption, and using drugs during part or the entire pregnancy. More specifications on exposures can be found in (Appendix I).

##### 2.3.2.2 Interpersonal factors

We evaluated women who experienced autonomy in decision-making during labour and birth using the valid Mothers' autonomy in decision-making (MADM) [18,32]. This measure consists of seven items that gauge a mother's involvement in deciding her maternity care plan [18]. Responses were rated on a 6-point Likert scale from 1 (strongly disagree) to 6 (strongly agree), resulting in a cumulative scale score ranging from 7 to 42. A higher score indicates a heightened sense of autonomy [18]. Similarly, we evaluated women's perceived respect using the valid Mothers On Respect index (MORi), comprising 14 items reflecting cultural and personal preferences, comfort levels, and potential barriers arising from language, race, religion, gender identity, or ethnicity [18,32]. Each item was rated on a 6-point Likert scale from 1 (strongly disagree) to 6 (strongly agree), generating a total scale score between 14 and 84. A higher score denotes a stronger perception of respect experienced by the mother [18]. Finally, we assessed partner and family support. More specifications on exposures can be found in (Appendix I).

##### 2.3.2.3 Organisational factors

We examined place of birth (at the hospital/obstetrician-clinical midwife, at the hospital/primary midwife, or in birth centres/home). During the COVID-19 pandemic in 2020, the number of women opting for home births increased compared the previous year [33]. Therefore, we considered period of birth, noting whether women participated in the study had their childbirth before 2020 (pre-pandemic) or from 2020 onwards (pandemic). We assessed continuity of care within the healthcare system using the Nijmegen Continuity of Care questionnaire (NCQ), a validated instrument designed to gauge an individual's perceived continuity of care [23]. It has two subscales; healthcare provider knows me (NCQ-1), and healthcare provider shows commitment (NCQ-2). Scoring and categorisation of NCQ subscales are available in (Appendix I).

### 2.4 Statistical analysis

Descriptive statistics were used to report relevant individual factors among the included and excluded populations (Table 1). Additionally, individual, interpersonal, and organisational factors among the included population and women who differed in positive/negative childbirth experience, were reported using Chi-Square or Fisher Exact tests where appropriate (Table 2).

**Table 1:** Demographic characteristics of study population groups, stratified by completing the CEQ2.0 questionnaire.

	Total population N = 1478 (100 %)	Included population Complete CEQ2.0 questionnaire N = 1141 (77.2 %)	Excluded population Incomplete CEQ2.0 questionnaire N = 337 (22.8 %)	Statistical differences between included and excluded population
	N (%)	N (%)	N (%)	P-value^a^
**INDIVIDUAL FACTORS**				
**Maternal age (in years)**				0.32
Below 30	377 (25.5)	284 (24.9)	93 (27.6)	
Above 30	1101 (74.5)	857 (75.1)	244 (72.4)	
**Marital status**				0.64
Single	60 (4.0)	48 (4.2)	12 (3.6)	
In a relationship	1418 (96.0)	1093 (95.8)	325 (96.4)	
**Sexual Orientation**				0.51
Heterosexual	1379 (93.3)	1062 (93.1)	317 (94.1)	
LGBTQIA+^b^	86 (5.8)	69 (6.0)	17 (5.0)	
Missing	13 (0.9)	10 (0.9)	3 (0.9)	
**Educational level**				**≤0.001**
Low	48 (3.2)	31 (2.7)	94 (27.9)	
Middle	332 (22.5)	238 (20.9)	17 (5.0)	
High	1092 (73.9)	868 (76.0)	224 (66.5)	
Missing	6 (0.4)	4 (0.4)	2 (0.6)	
**Income**				**0.03**
Low	106 (7.2)	81 (7.0)	25 (7.4)	
Middle	211 (14.3)	158 (13.8)	53 (15.7)	
High	991 (67.0)	785 (69.0)	206 (61.1)	
Not willing to share	151 (10.2)	104 (9.1)	47 (14.0)	
Missing	19 (1.3)	13 (1.1)	6 (1.8)	
**Ethnicity**				0.82
Dutch	1159 (78.4)	891 (78.1)	268 (79.6)	
Children of one or two migrants	92 (6.2)	73 (6.4)	19 (5.6)	
Migrants	227 (15.4)	177 (15.5)	50 (14.8)	
**Parity**				**0.03**
Nulliparous	409 (27.7)	300 (26.3)	109 (32.3)	
Multiparous	1069 (72.3)	841 (73.7)	228 (67.7)	
**Gestational age (in weeks)**				0.57
Preterm (<37)	117 (7.9)	86 (7.5)	31 (9.2)	
Early and Full term (37-40)	1029 (69.6)	800 (70.1)	229 (68.0)	
Late-term (>40)	332 (22.5)	255 (22.4)	77 (22.8)	
**Mode of birth**				**0.002**
Spontaneous vaginal	1070 (72.4)	853 (74.7)	217 (64.4)	
Instrumental vaginal	128 (8.7)	90 (7.9)	38 (11.3)	
Caesarian section-elective	97 (6.6)	66 (5.8)	31 (9.2)	
Caesarian section-emergency	183 (12.4)	132 (11.6)	51 (15.1)	

a Values in bold are statistically significant at p ≤ 0.05.

b LGBTQIA refers to Lesbian, Gay, Bisexual, Transgender, Queer, Intersex, and Asexual, and “+” stands for all of the other identities not included in the acronym.

**Table 2:** Individual, Interpersonal and Organisational factors of the total population and stratified for women with positive and negative childbirth experiences.

	Included population N = 1141	Population reported positive childbirth experience N = 856 (75 %)	Population reported negative childbirth experience N = 285 (25 %)	Statistical differences between populations with positive and negative childbirth experiences
	N (%)	N (%)	N (%)	P-value a
**INDIVIDUAL FACTORS**				
**Maternal age (in years)**				0.26
Below 30	284 (24.9)	206 (24.0)	78 (27.4)	
Above 30	857 (57.1)	650 (76.0)	207 (72.6)	
**Marital status**				0.99
Single	48 (4.2)	36 (4.2)	12 (4.2)	
In a relationship	1093(95.8)	820 (95.8)	273 (95.8)	
**Sexual Orientation**				0.09
Heterosexual	1062 (93.1)	804 (93.9)	258 (90.5)	
LGBTQIA+	69 (6.0)	46 (5.4)	23 (8.1)	
Missing	10 (0.9)	6 (0.7)	4 (1.4)	
**Educational level**				0.79
Low	31 (2.7)	24 (2.8)	7 (2.5)	
Middle	238 (20.9)	182 (21.3)	56 (19.6)	
High	868 (76.0)	647 (75.6)	221 (77.5)	
Missing	4 (0.4)	3 (0.3)	1 (0.4)	
**Income**				0.74
Low	81 (7.1)	63 (7.45)	18 (6.3)	
Middle	158 (13.9)	114 (13.3)	44 (15.4)	
High	785 (68.8)	591 (69.0)	194 (68.1)	
Not willing to share	104 (9.1)	80 (9.35)	24 (8.4)	
Missing	13 (1.1)	8 (0.9)	5 (1.8)	
**Ethnicity**				0.12
Dutch	891 (78.1)	679 (79.3)	212 (74.4)	
Children of one or two migrants	73 (6.4)	55 (6.4)	18 (6.3)	
Migrants	177 (15.5)	122 (14.3)	55 (19.3)	
**Parity**				**≤0.001**
Nulliparous	300 (26.3)	201 (23.5)	99 (34.7)	
Multiparous	841 (73.7)	655 (76.5)	186 (65.3)	
**Gestational age (in weeks)**				**≤0.001**
Preterm (<37)	86 (7.5)	51 (6.0)	35 (12.3)	
Early and Full term (37-40)	800 (70.1)	619 (72.3)	181 (63.5)	
Late term (>40)	255 (22.4)	186 (21.7)	69 (24.2)	
**Pregnancy complications**				**≤0.001**
Yes	733 (64.2)	507 (59.2)	226 (79.3)	
No	408 (35.8)	349 (40.8)	59 (20.7)	
**Mode of birth**				**≤0.001**
Spontaneous vaginal	853 (74.7)	707 (82.6)	146 (51.2)	
Instrumental vaginal	90 (7.9)	36 (4.2)	54 (19.0)	
Caesarean section elective	66 (5.8)	54 (6.3)	12 (4.2)	
Caesarean section emergency	132 (11.6)	59 (6.9)	73 (25.6)	
**Adverse lifestyle behaviours**				0.92
Yes	55 (4.8)	41 (4.8)	14 (4.9)	
No	1086 (95.2)	815 (95.2)	271 (95.1)	
**Adverse mental health**				**≤0.001**
Yes	299 (26.2)	205 (24.0)	94 (33.0)	
No	806 (70.6)	630 (73.6)	176 (61.7)	
Missing	36 (3.2)	21 (2.4)	15 (5.3)	
**INTERPERSONAL FACTORS**				
**Autonomy-MADM**^b^				**≤0.001**
Low	311 (27.3)	123 (14.4)	188 (66.0)	
Average	296 (25.9)	228 (26.6)	68 (23.8)	
High	534 (46.8)	505 (59.0)	29 (10.2)	
**Respect-MORi**^c^				**<0.001**
Low	92 (8.0)	10 (1.2)	82 (28.8)	
Average	245 (21.5)	113 (13.2)	132 (46.3)	
High	804 (70.5)	733 (85.6)	71 (24.9)	
**Partner support**				0.48
Low	30 (2.6)	21 (2.4)	9 (3.1)	
Neutral	109 (9.6)	76 (8.9)	33 (11.6)	
High	953 (83.5)	723 (84.5)	230 (80.7)	
No partner	49 (4.3)	36 (4.2)	13 (4.6)	
**Social support**				**≤0.001**
Low	84 (7.3)	54 (6.3)	30 (10.5)	
Neutral	251 (22.0)	171 (20.0)	80 (28.0)	
High	797 (69.9)	623 (72.8)	174 (61.1)	
Missing	9 (0.8)	8 (0.9)	1 (0.4)	
**ORGANISATIONAL FACTORS**				
**Period of birth**^d^				0.19
Pre-pandemic	318 (27.9)	230 (26.9)	88 (30.9)	
Pandemic	823 (72.1)	626 (73.1)	197 (69.1)	
**Place of birth**				**≤0.001**
Hospital/an obstetrician-a clinical midwife	723 (63.4)	465 (54.3)	258 (90.5)	
Hospital/primary care midwife	140 (12.3)	125 (14.6)	15 (5.3)	
Birth centres/home	278 (24.3)	266 (31.1)	12 (4.2)	
**Continuity of care** **Healthcare provider knows me NCQ-1**^e^				**≤0.001**
Low	444 (38.9)	236 (27.6)	208 (73)	
Average	409 (35.9)	359 (41.9)	50 (17.5)	
High	288 (25.2)	261 (30.5)	27 (9.5)	
**Continuity of care** **Healthcare provider shows commitment NCQ-2**^f^				**≤0.001**
Low	291 (25.5)	129 (15.1)	162 (56.8)	
Average	446 (39.1)	362 (42.3)	84 (29.5)	
High	404 (35.4)	365 (42.6)	39 (13.7)	

a Values in bold are statistically significant at p ≤ 0.05.

b MADM refers to The Mother's Autonomy in Decision Making (MADM) Scale.

c MORi refers to The Mothers on Respect (MORi) index.

d Pre-pandemic (years 2017–2019), Pandemic (years 2020–2022).

e NCQ-1 refers to Nijmegen Continuity Questionnaire subscale-1.

f NCQ-2 refers to Nijmegen Continuity Questionnaire subscale-2.

Next, we developed univariable and multivariable logistic models to examine associations of individual, interpersonal, and organisational factors with negative childbirth experience. In the univariable models, we assessed the crude association between each factor with negative childbirth experience. Upon modelling, associations were adjusted for factors present in each model. Model I tested individual factors, while, Model II tested interpersonal factors, to achieve this, we built two versions A and B; based on MADM and MORi, due to the high correlation between these two measures (Spearman's rho = 0.70). Model III tested organisational factors using versions A, and B, where we needed to base a model for NCQ-1, and NCQ-2, due to the high correlation between NCQ-1 and NCQ-2 (Spearman's rho = 0.75). High correlations between MADM and MORi, or NCQ-1 and NCQ-2 were also proven by previous literature [18,23]. Lastly, we gathered all factors in one model with four versions to avoid multicollinearity; resulting in Model IV A (MADM-NCQ-1), Model IV B (MORi-NCQ-1), Model IV C (MADM-NCQ-2), and Model IV D (MORi- NCQ-2). All models are displayed in (Table 3), where crude and adjusted associations were reported with odds ratio (OR) with accompanying 95 % Confidence Intervals (95 % CI). All data preparation and analyses were conducted by Statistics 28.0 (SPSS Inc., Chicago, IL). The level of significance was set at p ≤ 0.05.

**Table 3:** Associations of Individual, Interpersonal, and Organisational factors with Negative Childbirth Experience using socio-ecologial modelling.

	Bivariate relationship with negative childbirth experience	Model I All individual factors	Model II A All Interpersonal factors incl. MADM	Model II B All Interpersonal factors incl. MORi	Model III A All Organisational factors incl. NCQ-1	Model III B All Organisational factors incl. NCQ-2	Model IV A all factors incl. MADM and NCQ-1	Model IV B all factors incl. MORi and NCQ-1	Model IV C all factors incl. MADM and NCQ-2	Model IV D all factors incl. MORi and NCQ-2
	OR^a^ (95 % CI)	OR^a^ (95 % CI)	OR^a^ (95 % CI)	OR^a^ (95 % CI)	OR^a^ (95 % CI)	OR^a^ (95 % CI)	OR^a^ (95 % CI)	OR^a^ (95 % CI)	OR^a^ (95 % CI)	OR^a^ (95 % CI)
**INDIVIDUAL FACTORS**										
**Maternal age (in years)**										
<30	Ref.	Ref.					Ref.	Ref.	Ref.	Ref.
>30	0.84[0.62–1.14]	0.98[0.94–1.02]					0.78[0.50–1.20]	0.95[0.58–1.56]	0.75[0.48–1.16]	0.91[0.56–1.49]
**Marital status**^b^										
Spouse/partner	Ref.	Ref.					NA^b^	NA^b^	NA^b^	NA^b^
No spouse/partner	1.00[0.51–1.95]	0.76[0.34–1.70]								
**Sexual orientation**										
Heterosexual	Ref.	Ref.					Ref.	Ref.	Ref.	Ref.
LGBTQIA+	1.55[0.92–2.62]	1.37[0.77–2.44]					1.11[0.51–2.43]	0.97[0.38–2.46]	1.09[0.50–2.37]	0.92[0.37–2.28]
**Education level**										
Middle	Ref.	Ref.					Ref.	Ref.	Ref.	Ref.
Low	0.94[0.39–2.31]	1.02[0.39–2.67]					2.40[0.77–7.43]	**3.65[1.03**–**12.9]**	2.31[0.75–7.14]	3.34[0.96–11.63]
High	1.11[0.80–1.55]	1.13[0.76–1.68]					1.66[1.00–2.78]	**1.93[1.09**–**3.43]**	**1.71[1.02**–**2.88]**	**2.00[1.12**–**3.58]**
**Income**										
Middle	Ref.	Ref.					Ref.	Ref.	Ref.	Ref.
Low	0.74[0.38–1.38]	0.74[0.37–1.49]					0.64[0.25–1.65]	0.76[0.28–2.03]	0.66[0.26–1.67]	0.81[0.30–2.20]
High	0.85[0.57–1.24]	0.74[0.48–1.30]					0.65[0.37–1.14]	0.79[0.43–1.47]	0.67[0.38–1.17]	0.81[0.43–1.49]
Not willing to share	0.77[0.43–1.37]	0.73[0.38–1.38]					0.54[0.24–1.22]	0.66[0.26–1.64]	0.54[0.24–1.22]	0.66[0.27–1.65]
**Ethnicity**										
Dutch	Ref.	Ref.					Ref.	Ref.	Ref.	Ref.
Children of one or two migrants	1.04[0.60–1.82]	1.11[0.61–2.01]					1.03[0.47–2.25]	1.38[0.61–3.09]	1.03[0.47–2.26]	1.41[0.63–3.14]
Migrants	**1.44[1.01**–**2.05**] c	1.38[0.92–2.08]					1.53[0.91–2.58]	1.08[0.61–1.91]	1.44[0.85–2.44]	1.02[0.58–1.82]
**Parity**										
Multiparous	Ref.	Ref.					Ref.	Ref.	Ref.	Ref.
Nulliparous	**1.73[1.30**–**2.31]**	1.33[0.96–1.84]					0.92[0.60–1.40]	0.92[0.58–1.45]	0.90[0.59–1.37]	0.89[0.56–1.40]
**Gestational age (in weeks)**										
Early and Full term (37-40)	Ref.	Ref.					Ref.	Ref.	Ref.	Ref.
Preterm (<37)	**2.34[1.48**–**3.72]**	**1.82[1.11**–**2.97]**					0.80[0.43–1.50]	1.11[0.57–2.16]	0.83[0.44–1.56]	1.10[0.56–2.15]
Late term (>40)	1.26[0.91–1.75]	1.18[0.82–1.68]					0.98[0.62–1.54]	0.88[0.52–1.48]	1.01[0.64–1.59]	0.93[0.56–1.55]
**Pregnancy complications**	**2.63[1.92**–**3.62]**	2.69[1.90–3.81]					1.36[0.86–2.15]	1.30[0.79–2.15]	1.38[0.86–2.19]	1.37[0.83–2.27]
**Mode of birth**										
Spontaneous vaginal	Ref.	Ref.					Ref.	Ref.	Ref.	Ref.
Instrumental vaginal	**7.26[4.59**–**11.4]**	**6.44[3.92**–**10.57]**					**3.71[2.04**–**6.73]**	**3.35[1.76**–**6.40]**	**4.08[2.24**–**7.43]**	**3.84[2.01**–**7.35]**
Caesarean section elective	1.07[0.56–2.06]	1.09[0.56–2.11]					0.86[0.39–1.92]	0.71[0.30–1.69]	0.79[0.35–1.76]	0.60[0.25–1.43]
Caesarean section emergency	**5.99[4.07**–**8.82]**	**4.97[3.27**–**7.55]**					**4.13[2.43**–**7.02]**	**4.40[2.43**–**7.91]**	**4.06[2.38**–**6.92]**	**4.23[2.35**–**7.63]**
**Adverse lifestyle behaviours**	1.02[0.55–1.91]	0.90[0.45–1.78]					0.53[0.22–1.27]	0.68[0.26–1.80]	0.50[0.21–1.20]	0.60[0.23–1.58]
**Adverse mental health**	**1.64[1.22**–**2.20]**	**1.43[1.04**–**1.97]**					**1.60[1.05**–**2.45]**	1.34[0.85–2.12]	**1.59[1.03**–**2.43]**	1.32[0.83–2.09]
**INTERPERSONAL FACTORS**										
**Autonomy-MADM**^d^	**0.86[0.84**–**0.88]**		**0.86[0.84**–**0.87]**	NA			**0.88[0.86**–**0.90]**	NA	**0.88[0.86**–**0.90]**	NA
**Respect-MORi**^e^	**0.86[0.84**–**0.87]**		NA	**0.86[0.84**–**0.87]**			**NA**	**0.87[0.85**–**0.88]**	**NA**	**0.87[0.85**–**0.89]**
**Partner support**										
Neutral	Ref.		Ref.	Ref.			Ref.	Ref.	Ref.	Ref.
Low	0.99[0.40–2.38]		1.15[0.38–3.44]	1.21[0.33–4.41]			1.34[0.38–4.74]	1.93[0.46–8.01]	1.24[0.35–4.33]	1.77[0.44–7.00]
High	0.73[0.47–1.13]		0.88[0.49–1.56]	1.73[0.89–3.36]			0.89[0.44–1.79]	1.63[0.74–3.57]	0.85[0.42–1.72]	1.55[0.70–3.41]
No partner	0.83[0.39–1.76]		0.55[0.20–1.47]	0.90[0.30–2.73]			0.41[0.12–1.35]	0.67[0.17–2.56]	0.35[0.10–1.18]	0.56[0.14–2.15]
**Social support**										
Neutral	Ref.		Ref.	Ref.			Ref.	Ref.	Ref.	Ref.
Low	1.18[0.70–1.99]		1.63[0.85–3.11]	1.52[0.76–3.06]			1.03[0.46–2.27]	1.16[0.49–2.73]	0.99[0.44–2.19]	1.05[0.45–2.47]
High	**0.59[0.43**–**0.81]**		0.69[0.46–1.03]	0.72[0.46–1.12]			**0.04[0.61-0.39]**	0.60[0.36–1.01]	**0.61[0.38**–**0.97]**	**0.59[0.36**–**0.99]**
**ORGANISATIONAL FACTORS**										
Pre-pandemic	Ref.				Ref.	Ref.	Ref.	Ref.	Ref.	Ref.
Pandemic	0.82 [0.61–1.10]				0.86[0.62–1.2]	0.86[0.61–1.2]	1.06[0.70–1.61]	0.93[0.59–1.47]	1.07[0.70–1.62]	0.94[0.60–1.48]
**Place of birth**										
Hospital/an obstetrician-clinical midwife	Ref.				Ref.	Ref.	Ref.	Ref.	Ref.	Ref.
Hospital/primary care midwife	**0.21[0.12**–**0.37]**				0.58[0.31–1.06]	**0.46[0.25**–**0.83]**	0.91[0.41–2.02]	1.01[0.43–2.39]	0.89[0.41–1.91]	0.84[0.36–1.91]
Birth centres/home	**0.08[0.04**–**0.14]**				**0.22[0.12**–**0.43]**	**0.18[0.09**–**0.34]**	0.51[0.24–1.09]	0.69[0.31–1.55]	0.49[0.23–1.02]	0.55[0.25–1.21]
**Healthcare provider knows me NCQ-1**^f^	**0.40[0.35**–**0.47]**				**0.49[0.42**–**0.58]**	NA	**0.77[0.62**–**0.94]**	**0.66[0.53**–**0.82]**	NA	NA
**Healthcare provider shows commitment NCQ-2**^g^	**0.42[0.37**–**0.47]**				NA	**0.50[0.44**–**0.57]**	NA	NA	**0.72[0.60**–**0.86]**	**0.69[0.57**–**0.83]**

a Associations in bold are statistically significant at p <0.05.

b Models included Partner support did not include Marital status.

c Associations in bold are statistically significant at p <0.05.

d MADM refers to The Mother's Autonomy in Decision Making (MADM) Scale.

e MORi refers to The Mothers on Respect (MORi) index.

f NCQ-1 refers to Nijmegen Continuity Questionnaire subscale-1.

g NCQ-2 refers to Nijmegen Continuity Questionnaire subscale-2.

## 3 Results

### 3.1 Sample characteristics

Between May and December 2022, approximately 1905 individuals initiated the online BESt-NL survey. Among them, 427 were subsequently excluded for various reasons (Fig. 1). Ultimately, a total of 1478 eligible women, constituting 78 % of the initial population, participated in the BESt-NL study (Fig. 1).

First, we compared differences in individual factors among women who were included, the population who completed the CEQ2.0 questionnaire: 77.2 % (n = 1141), and the population who did not complete the CEQ2.0 questionnaire and were excluded from our study: 22.8 % (n = 337). Compared to the included population, the excluded population had a higher percentage of low education (5 % vs. 2.7 %), low income (7.4 % vs. 7 %), nulliparous women (32.3 % vs. 26.3 %) and more emergency CS (15.1 % vs. 11.6 %), with no significant differences observed in other individual factors (Table 1).

Within the included population, factors within the individual level were as follows: 57.1 % of maternal age was over 30 years, 15.5 % were migrants, and 26.2 % had adverse mental health. Non-spontaneous vaginal births (Instrumental vaginal, elective CS, emergency CS) constituted 25.3 % of the sample. At the interpersonal level, 27.3 % of women reported low autonomy and 8 % reported low respect. At the organisational level, 63.4 % of women gave birth in a hospital, while a relevant fraction reported low continuity of care in both NCQ-1, and NCQ-2 (38.9 %, and 25.5 %, respectively) (Table 2).

### 3.2 Differences between women with positive or negative childbirth experiences on individual, interpersonal, and organisational factors

In the included population (n = 1141), 75 % (n = 856) of women had positive birth experience whereas 25 % (n = 285) of women had negative childbirth experience. Women with negative childbirth experience had statistically significant differences in parity; nulliparous (34.7 % vs 23.5 %), gestational age; preterm births (12.3 % vs 6 %), pregnancy complications (79.3 % vs 59.2 %), mode of birth; CS emergency (25.6 % vs 6.9 %), and adverse mental health (33 % vs 24 %). They had higher percentages of low autonomy (66.0 % vs 14.4 %), low respect (28.8 % vs 1.2 %), and low social support (10.5 % vs 6.3 %). Lastly, they gave birth within obstetrician-clinical midwife-led care (90.5 % vs 54.3 %), and they reported higher percentages of low continuity of care in NCQ-1 (73 % vs 27.6 %), and NCQ-2 (56.8 % vs 15.1 %) (Table.2).

### 3.3 The association of individual, interpersonal and organisational factors with negative childbirth experience

Univariable logistic regression resulted in women of migration background, nulliparity, preterm births, pregnancy complications, instrumental vaginal births, emergency CS, and adverse mental health having a higher likelihood of experiencing negative childbirth. Whereas the likelihood of negative childbirth was decreased by each unit increase in autonomy, respect, and high social support. Also, mode of birth as giving birth under hospital/primary care midwife, and birth centres/home decreased the odds of negative childbirth experience. Lastly, the likelihood of negative childbirth experience was decreasing by each unit increase in continuity of care in NCQ-1, and NCQ-2 (Table 3).

Upon adding all individual factors in Model I, significant association with negative childbirth experience persisted in preterm births, pregnancy complications, instrumental vaginal births, emergency CS, and adverse mental health (OR 1.64, 95 % CI: 1.22–2.20). In models representing interpersonal level, Models II (autonomy; A), (respect; B), each unit increase in autonomy and respect remained inversely associated with negative childbirth experience. In models representing organisational level, Models III (NCQ-1; A, (NCQ2; B), Model III A showed mode of birth as giving birth at a birth centre/home exhibited an inverse association with negative childbirth experience, while in Model III B, mode of birth in both categories (hospital/primary midwife, at a birth centre/home) remained inversely associated with negative childbirth experience. Continuity of care in NCQ-1, and NCQ-2 within organisational models, Models III (NCQ-1; A, (NCQ2; B) remained inversely associated with negative childbirth experience (Table 3).

Finally, we added all factors in one model, Model IV, with four versions according to the measures included in the models; MADM, MORi, NCQ-1, and NCQ-2. In Model IV A, instrumental vaginal birth, emergency CS, and adverse mental health remained associated with negative childbirth experience. Each unit increase in autonomy and high social support remained inversely associated. Similarly, each unit increase in continuity of care in NCQ-1 remained inversely associated (Table 3).

Model IV B had an emerging significance in education: low education (OR 3.65, 95 % CI: 1.03–12.9) and high education (OR 1.93, 95 % CI: 1.09–3.43). Meanwhile, instrumental vaginal birth and emergency CS remained associated with negative childbirth experience. Whereas, each unit increase in respect remained inversely associated. Similarly, each unit increase in continuity of care NCQ-1 remained inversely associated (Table 3).

Model IV C had only the high education category (OR 1.71, 95 % CI:1.02–2.88), along with instrumental vaginal birth, emergency CS, and adverse mental health as factors associated with negative childbirth experience. Whereas, each unit increase in autonomy, in addition to, high social support remained inversely associated. Similarly, each unit increase in continuity of care in NCQ-2 remained inversely associated ().

In Model IV D, high education remained associated with negative childbirth along with instrumental vaginal birth, and emergency CS. Whereas, each unit increase in respect and high social support remained inversely associated with negative childbirth experience. Similarly, each unit increase in continuity of care in NCQ-2 remained inversely associated (Table 3).

**Fig. 3:**
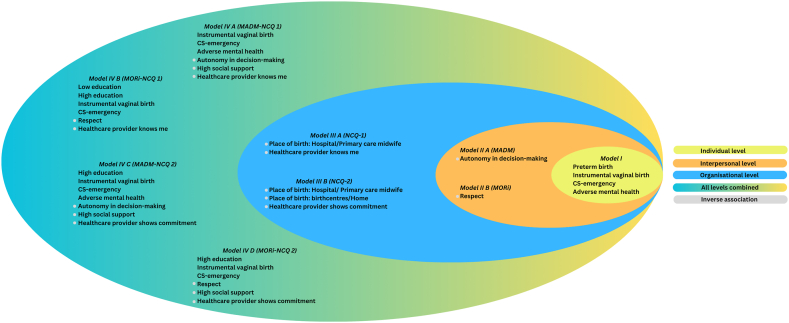
Significant associations with negative childbirth experience.

The Nagelkerke R^2^, which measures how much the model explains the variance in negative childbirth experience on a scale from 0 to 1, showed the following values: Model IV A had 0.50, Model IV B had 0.60, Model IV C had 0.51, and Model IV D had 0.60. All significant factors among various models are visualized in (Fig. 3).

## 4 Discussion

In this study, we aimed to examine the occurrence of negative childbirth experiences by comparing women who reported positive versus negative experiences using the validated CEQ2.0 questionnaire. Additionally, inspired by the WHO framework for high-quality intrapartum care and previous literature, we sought to examine factors associated with negative childbirth experiences by building socio-ecological model that categorised these factors based on their level of influence (individual, interpersonal, and organisational factors).

### 4.1 Differences between women who report positive and negative childbirth experience

25 % of women (n = 1141) reported negative childbirth experience, and on the individual level, they reported statistically significant differences in factors of pregnancy characteristics i.e., nulliparity, preterm birth, pregnancy complications, and non-spontaneous birth such as instrumental vaginal and emergency CS, as well as, in pre-existing adverse mental health, which aligns with previous studies [3,6,30]. Unlike previous studies, non-significant results in sociodemographics i.e., education, income, sexual orientation could reflect a potential non-discriminatory experience of women in our study [8,11,12]. Factors we hypothesised and included in the interpersonal level were predominantly significant. Women with negative childbirth experience reported low autonomy, respect, and social support, which aligns with the 10.13039/100004423WHO framework and previous studies [4,18]. Similarly, factors included in the organisational level were all significant, except for period of birth. Women with negative childbirth experience mostly underwent obstetrician-clinical midwife-led care, and experienced lower continuity of care, which aligns with previous studies [19,21].

### 4.2 Factors associated with negative childbirth experience utilising socio-ecological modelling approach

Upon univariable logistic regression, we found significant associations between negative childbirth experience and factors we anticipated based on the results of the first aim (i.e., nulliparity, preterm births, pregnancy complications, instrumental vaginal births, emergency CS, and adverse mental health), and newly added factors (i.e., ethnicity). Additionally, we found significant inverse associations with high autonomy, high respect, high social support, and high continuity of care.

### 4.3 Socio-ecological models with defined levels of influence

Modelling factors into levels of defined influence has affected the significance of some factors. Model I of individual factors kept significant associations in pregnancy characteristics (preterm birth, instrumental vaginal, and emergency CS), in addition to, adverse mental health. Whereas, ethnicity and parity have lost their significance upon modelling. In contrast, modelling did not affect the significance of interpersonal factors (Model II A, Model II B) and organisational factors (Model III A, Model III B), with the exception of social support. Notably, we found continuity of care subscale might influence the significance of place of birth categories, as healthcare provider knows me (NCQ-1), the category of birth centres/home was significant, while healthcare provider shows commitment (NCQ-2), both categories' hospitals/primary midwives and birth centres/home were significant. This could be explained by women experiencing less continuity of care when referred from primary care to secondary care settings [20].

### 4.4 Robust, emerged or diminished associations upon adding all factors into one model

In models that included all factors, socio-ecological model approach helped us uncover associations that were previously overlooked when tested separately. Additionally, robust associations with negative childbirth experiences remained significant after adjusting for other factors. Education became significant when we applied socio-ecological model approach. The significance of low education aligns with previous literature [3,6], and high education category suggests women of high education report less satisfaction with healthcare [34]. Robust associations were found in pregnancy characteristics. Instrumental vaginal births and emergency CS remained significant in all models, while adverse mental health remained significant in autonomy-based models but not in respect-based models. This difference might be due to a mediating effect of respect on the pathway between adverse mental health and negative childbirth experiences. Previous studies suggest that women with pre-existing mental health issues are often referred to emergency care in hospitals due to poor antenatal care attendance, which could explain this mediation effect [15]. However, more research is needed to examine how respect could affect the relationship between women with adverse mental health and negative childbirth experience.

Robust associations were found in interpersonal factors, as autonomy and respect remained significant in all models, which reflects the necessity of the WHO framework of high-quality intrapartum care that emphasises non-medical aspects of care i.e., effective communications, respectful care, and inclusion of women's preferences [4]. Additionally, socio-ecological model approach demonstrated that social support was non-significant when tested within the interpersonal level, however, upon adding all factors from all levels into one model, it became significant. This reflects the need for socio-ecological model approach in addressing complex health events to avoid overlooking essential modifiable factors [9].

Robust associations were found in organisational factors, as continuity of care in both subscales (NCQ-1, NCQ-2) remained significant in all models, which augments the WHO framework [4], and emphasises previous study results [21]. The socio-ecological approach suggested that the place of birth, when tested separately within organisational models, was significant. However, upon adding all factors into one model, it lost its significance. This suggests that the place of birth is related to other factors, such as referrals from primary midwife-led care to obstetrician/clinical midwife-led care due to pregnancy complications [18], intrapartum interventions [35], or lack of planning place of birth beforehand, the latter case lies within concepts of autonomy and respect [16,35].

### 4.5 Strengths and limitations

To the best of our knowledge, this is the first study to design socio-ecological model of Dutch women's childbirth experiences in the realm of high-quality intrapartum care research.

We examined various factors that influence childbirth experience on different levels. At the individual level, we considered women's sociodemographics, pregnancy characteristics, adverse lifestyle behaviours, and adverse mental health. Women's factors within the interpersonal level: autonomy in decision-making, respect, and partner and social support. Women's factors within the organisational level: place of birth, period of birth in relation to COVID-19, and continuity of care. Our sample embraced inclusivity in terms of language barrier as we created two versions of the survey; English and Dutch, and we were successful in including more migrants (15.4 %), and more diverse sexual orientations (LGBTQIA+; 5.8 %) compared with other Dutch surveys [8,18]. Additionally, we included validated measures in the Dutch setting regarding childbirth experience (CEQ2.0), autonomy (MADM), respect (MORi) and continuity of care (NCQ) [18,23].

Our study has also some limitations. First, the retrospective design of the survey introduced the possibility of recall bias. However, previous research suggests that accurately reporting childbirth experiences may require time for reflection after the event [36]. Second, despite the higher diverse sample, the results are not generalisable to the Dutch population, however, our results are aligned with a recent national survey that recruited 12,239 women [8]. Third, the lower completion rate of the CEQ2.0 among women with lower education and income, higher nulliparity, and more emergency CS raises concerns about selection bias and the potential underestimation of the effect of these factors on negative childbirth experiences. Nevertheless, our study's non-response rates align with those observed in a previous study utilising CEQ2.0, which stated women of higher education had a higher response rate, with no significant differences in income levels and mode of birth [37].

### 4.6 Implications

Our study findings could inform initiatives targeting subgroups. This involves enhancing prenatal mental health care, autonomy in decision-making, respectful care, and recognizing the benefits of midwife-led care.

We examined the occurrence of adverse mental health within two years before or during pregnancy. In the Netherlands, women with existing mental health conditions continue receiving care from their psychologists, provided there are no severe psychiatric issues. In complex cases, the woman is referred to a Psychiatry, Obstetrics, and Paediatrics (POP) clinic [38]. Women without previous mental health support are referred by their general practitioner to a psychologist or practical mental healthcare assistant [38]. Therefore, we emphasise the importance of collaboration between primary healthcare centres and specialised mental healthcare to ensure early intervention and effective management of mental health issues. Migrant women in our study experienced a higher crude odds ratio of negative childbirth experiences in comparison with women of Dutch origin, in alignment with a recent national survey [39]. An initiative against negative childbirth experiences within subgroups could be the implementation of cultural competence programs for healthcare providers. These programs aim to enhance the ability to collaborate effectively with individuals from diverse cultural backgrounds, thereby improving both healthcare providers' experiences and women's health outcomes [40]. The significance of social support suggests healthcare providers inquire about the social networks of women planning pregnancy, particularly first-time mothers [2]. This study finds continuity of care as a fundamental element of midwife-led care, similar to previous findings [21]. Therefore, improving the midwife-led care model could involve addressing modifiable factors that cause referrals from midwife-led care to obstetrician-led care, such as screening for pregnancy complications, late-term births, and foetal positioning [18,41].

### 4.7 Future research

To further examine precursors of negative childbirth experience, future studies require more representative samples of subgroups. Techniques to increase participation include providing incentives such as gift cards, and utilising both online and offline survey methods, including paper surveys and in-person interviews, to ensure accessibility for individuals with limited internet access. Strategies to enhance accessibility involve disseminating surveys at postnatal care centres (Kraamzorg) and asking women to report their previous childbirth experiences [42]. Kraamzorg is covered by the Dutch basic health insurance which ensures the inclusivity of participants with low education, low income, adverse lifestyle behaviours during pregnancy, diverse ethnic backgrounds, and diverse sexual orientations.

## 5 Conclusion

In this study, we utilised socio-ecological model to examine the complex experience of childbirth among Dutch women using data from BESt-NL study. We examined factors associated with negative childbirth experiences that revealed factors that might be overlooked when tested separately such as education. Our results reflect the need to enhance efforts within primary healthcare settings to identify pre-existing mental health disorders among women planning pregnancy, and also called for mitigating ethnic differences by implementing cultural competence programs. This study aligns with the WHO framework for high-quality intrapartum care, emphasising the positive impact of high autonomy in decision-making and respect in reducing the likelihood of negative childbirth experiences. Additionally, it emphasised the critical role of high continuity of care in maternal health, noting its inverse association with negative childbirth experiences and suggesting that midwife-led care may contribute to this association.

## Funding information

We acknowledge funding from the Faculty of Economics and Business of the University of Groningen for the interdisciplinary PhD project “Smoking during pregnancy: the endgame”. This work was supported by the Dutch institute ZonMW (grant number 531 003018) as part of the broader project “Together we’ll quit smoking!” “Optimizing the implementation of the Trimbos guideline “smoking cessation counselling” in the daily practice of healthcare professionals supporting low-SES pregnant women in the North or the Netherlands.” The funding sources had no involvement in study design, data collection, analysis, or interpretation of data.

## CRediT authorship contribution statement

**Tamool A.S. Muhamed:** Conceptualization, Data curation, Formal analysis, Investigation, Methodology, Software, Validation, Visualization, Writing – original draft, Writing – review & editing. **Viola Angelini:** Conceptualization, Funding acquisition, Methodology, Project administration, Resources, Supervision, Validation, Writing – review & editing. **Laura Viluma:** Conceptualization, Funding acquisition, Methodology, Project administration, Resources, Supervision, Validation, Writing – review & editing. **Hazel RM. Keedle:** Data curation, Writing – review & editing. **L. Lilian Peters:** Conceptualization, Data curation, Funding acquisition, Investigation, Methodology, Project administration, Resources, Software, Supervision, Validation, Writing – review & editing.

## Declaration of competing interest

The authors declare that they have no known competing financial interests or personal relationships that could have appeared to influence the work reported in this paper.
