# Supplementary material for: Negative childbirth experience in Dutch women: A socio-ecological analysis of individual, interpersonal, and organisational factors from the birth experience study
Source: Heliyon. 2024 Dec 15;11(1):e41254. doi: 10.1016/j.heliyon.2024.e41254 (PMC11825256; doi:10.1016/j.heliyon.2024.e41254)
Supplement: Multimedia component 1 [file mmc1.docx]

**Appendix I**

**List of variables**

| Variable | Specification |
| --- | --- |
| Education | We recoded 10 responses of *education*;  (1) No education (primary or primary education not completed)  (2) Primary education (primary education, special primary education)  (3) Primary or pre-vocational education  (4) Secondary general secondary education, (5) Secondary vocational education or vocational training  (6) Higher general and pre-university education  (7) Higher vocational education  (8) Scientific education (university bachelor/master/PhD)  (9) Others  (10) I'd rather not answer this,  into three categories of;  1-Low education that covered the 1- 4 responses  2-Middle education included the 5- 6 responses  3-High education encompassed the 7- 8 responses, while 9-10 responses were recoded as missing values |
| Monthly Household Income | We categorized *monthly household income* into;  1-Low income of less than 1999 €  2-Middle income of up to € 2900  3-High income of more than € 3000  4-Participants who were not willing to report their income |
| Parity | 1-First childbirth (nulliparous)  2-Subsequent childbirth (multiparous) |
| Gestational age | The duration of the gestational age was following WHO-ICD-10 regulations but merged post-term within the late-term category;  1-Preterm (less than 37 weeks)  2-Full-term (37- 40 weeks)  3-Late-term (more than 40 weeks) |
| Mode of birth | 1-Spontaneous vaginal births  2-Instrumental vaginal birth with assisted forceps or vacuum  3- Elective caesarean section (CS)  4- Emergency CS |
| Pregnancy complications | “Were there any complications or risk factors during pregnancy and/or childbirth? Select all that apply.” Any selected answer were recoded as “Yes”  -High blood pressure  -Pregnancy Poisoning/HELPP  -Gestational Diabetes  -Bladder infection  -Vaginal yeast infection  -Sexual transmitted disease, namely;  -Other infectious diseases namely;  -Thrombosis  -Growth retardation baby  Blood loss during pregnancy in the weeks 0 -12 (first trimester)  Blood loss during pregnancy in the weeks 13 – 26 (second trimester)  -Blood loss during pregnancy in the weeks 27 – 42 (third trimester)  -Excessive blood loss immediately after the birth of your child (Fluxus/HPP)  -Premature contractions or ruptured membranes (before 37 weeks of pregnancy)  -Baby had pooped in amniotic fluid (meconium)  -Waters were broken for more than 24 hours  -Other complication |
| Adverse lifestyle behaviours | “Did you use alcohol, drugs or cigarettes during pregnancy?” |
| Adverse mental health | “In the two years before or during your pregnancy, were you depressed or anxious for more than 2 weeks?” |
| Mothers Autonomy in Decision-Making scale (MADM) | 1-Low autonomy (7-24)  2-Average autonomy (25-33)  3-High autonomy (34-42). |
| The Mothers on Respect index (MORi) | 1-Low respect (14-49)  2-Average (50-66)  3-High (67-84) |
| Partner and Social support | Rating the level of support before and during pregnancy.  1-Low  2-Neutral  3-High  4- No partner (for partner support variable) |
| Place of birth | We categorized place of birth into three categories;  1-At the hospital led by either obstetrician and/or clinical midwife  2-At the hospital led by their primary care midwives  3-At birth centres or homes  The last category contained birth centres (n=28), homes with midwives (n=286), and homes without midwives (n=8) |
| Continuity of care; Nijmegen Continuity Questionnaire; NCQ-1, NCQ-2 | This tool comprehensively evaluates various facets of continuity, including the presence of a dedicated care provider and the effectiveness of communication and collaboration among different care providers, independent of morbidity and across diverse care settings. The NCQ comprises two subscales: 'personal continuity: care provider knows me,' NCQ-1, consisting of 5 items, and 'personal continuity: care provider shows commitment,' NCQ-2, consisting of 3 items. Responses were rated on a 5-point Likert scale from 1 (totally disagree) to 5 (totally agree). The subscale scores were calculated as the mean of the items in each subscale. Each subscale eventually has a mean score;  1-Low; NCQ-scores < 2  2-Moderate; a mean score of 3  3-High; a mean score of 4 or higher |
